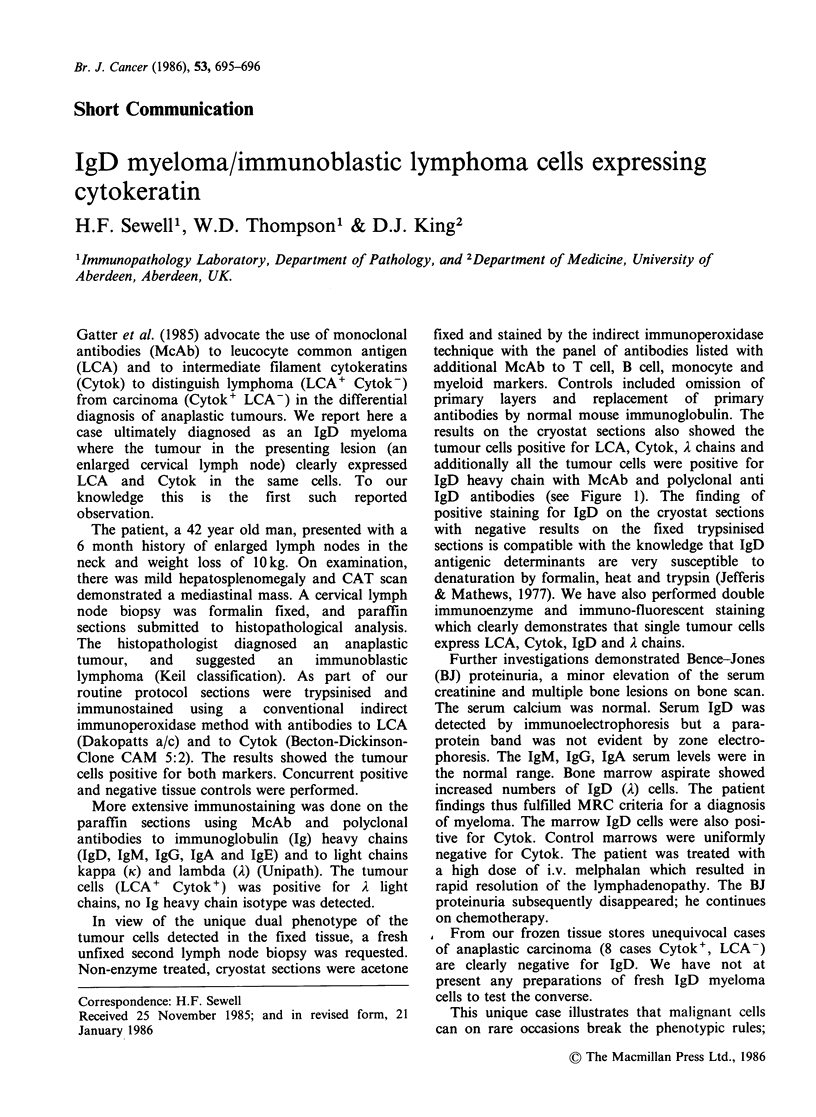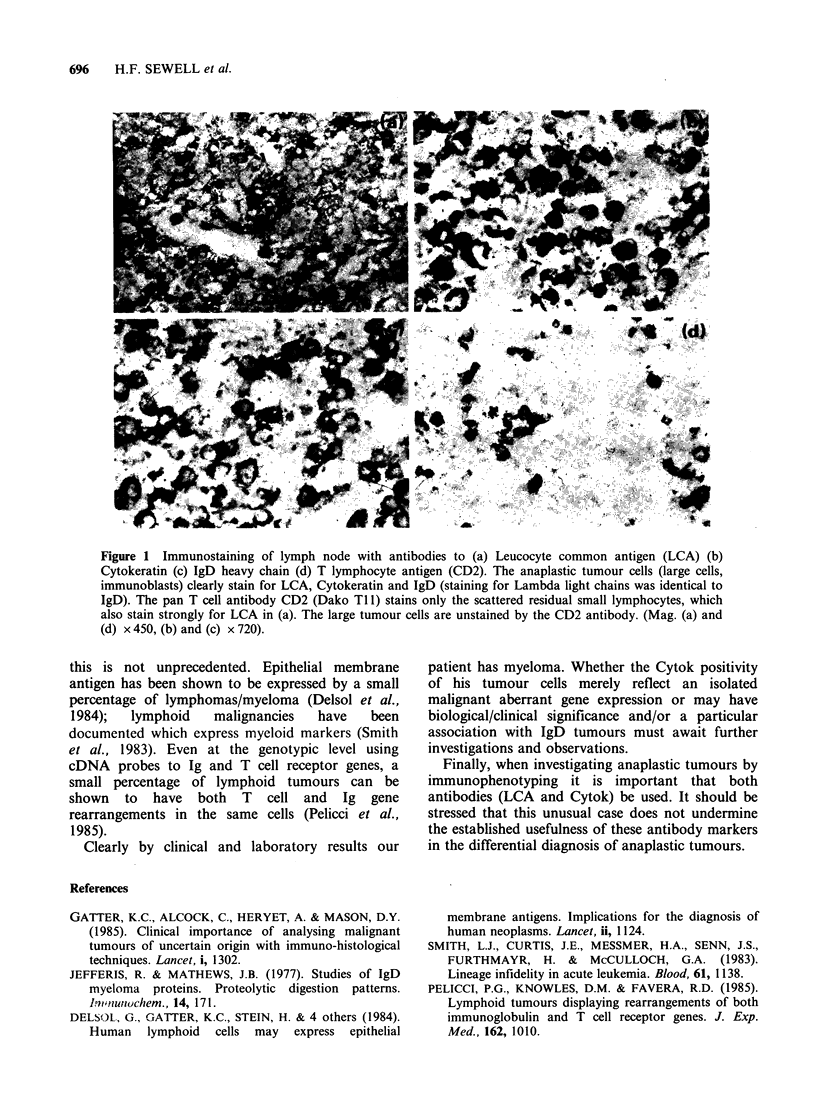# IgD myeloma/immunoblastic lymphoma cells expressing cytokeratin.

**DOI:** 10.1038/bjc.1986.115

**Published:** 1986-05

**Authors:** H. F. Sewell, W. D. Thompson, D. J. King

## Abstract

**Images:**


*Br. J. Cancer* (1986), **53**, 695–696

### Short Communication

### IgD myeloma/immunoblastic lymphoma cells expressing cytokeratin

H.F. Sewell[1], W.D. Thompson[1] & D.J. King[2]

[1]*Immunopathology Laboratory, Department of Pathology, and* [2]*Department of Medicine, University of Aberdeen, Aberdeen, UK.*

Gatter *et al.* (1985) advocate the use of monoclonal antibodies (McAb) to leucocyte common antigen (LCA) and to intermediate filament cytokeratins (Cytok) to distinguish lymphoma (LCA$^+$ Cytok$^-$) from carcinoma (Cytok$^+$ LCA$^-$) in the differential diagnosis of anaplastic tumours. We report here a case ultimately diagnosed as an IgD myeloma where the tumour in the presenting lesion (an enlarged cervical lymph node) clearly expressed LCA and Cytok in the same cells. To our knowledge this is the first such reported observation.

The patient, a 42 year old man, presented with a 6 month history of enlarged lymph nodes in the neck and weight loss of 10 kg. On examination, there was mild hepatosplenomegaly and CAT scan demonstrated a mediastinal mass. A cervical lymph node biopsy was formalin fixed, and paraffin sections submitted to histopathological analysis. The histopathologist diagnosed an anaplastic tumour, and suggested an immunoblastic lymphoma (Keil classification). As part of our routine protocol sections were trypsinised and immunostained using a conventional indirect immunoperoxidase method with antibodies to LCA (Dakopatts a/c) and to Cytok (Becton-Dickinson-Clone CAM 5:2). The results showed the tumour cells positive for both markers. Concurrent positive and negative tissue controls were performed.

More extensive immunostaining was done on the paraffin sections using McAb and polyclonal antibodies to immunoglobulin (Ig) heavy chains (IgD, IgM, IgG, IgA and IgE) and to light chains kappa ($\kappa$) and lambda ($\lambda$) (Unipath). The tumour cells (LCA$^+$ Cytok$^+$) was positive for $\lambda$ light chains, no Ig heavy chain isotype was detected.

In view of the unique dual phenotype of the tumour cells detected in the fixed tissue, a fresh unfixed second lymph node biopsy was requested. Non-enzyme treated, cryostat sections were acetone fixed and stained by the indirect immunoperoxidase technique with the panel of antibodies listed with additional McAb to T cell, B cell, monocyte and myeloid markers. Controls included omission of primary layers and replacement of primary antibodies by normal mouse immunoglobulin. The results on the cryostat sections also showed the tumour cells positive for LCA, Cytok, $\lambda$ chains and additionally all the tumour cells were positive for IgD heavy chain with McAb and polyclonal anti IgD antibodies (see Figure 1). The finding of positive staining for IgD on the cryostat sections with negative results on the fixed trypsinised sections is compatible with the knowledge that IgD antigenic determinants are very susceptible to denaturation by formalin, heat and trypsin (Jefferis & Mathews, 1977). We have also performed double immunoenzyme and immuno-fluorescent staining which clearly demonstrates that single tumour cells express LCA, Cytok, IgD and $\lambda$ chains.

Further investigations demonstrated Bence–Jones (BJ) proteinuria, a minor elevation of the serum creatinine and multiple bone lesions on bone scan. The serum calcium was normal. Serum IgD was detected by immunoelectrophoresis but a paraprotein band was not evident by zone electrophoresis. The IgM, IgG, IgA serum levels were in the normal range. Bone marrow aspirate showed increased numbers of IgD ($\lambda$) cells. The patient findings thus fulfilled MRC criteria for a diagnosis of myeloma. The marrow IgD cells were also positive for Cytok. Control marrows were uniformly negative for Cytok. The patient was treated with a high dose of i.v. melphalan which resulted in rapid resolution of the lymphadenopathy. The BJ proteinuria subsequently disappeared; he continues on chemotherapy.

From our frozen tissue stores unequivocal cases of anaplastic carcinoma (8 cases Cytok$^+$, LCA$^-$) are clearly negative for IgD. We have not at present any preparations of fresh IgD myeloma cells to test the converse.

This unique case illustrates that malignant cells can on rare occasions break the phenotypic rules;

Correspondence: H.F. Sewell
Received 25 November 1985; and in revised form, 21 January 1986

© The Macmillan Press Ltd., 1986

**Figure 1** Immunostaining of lymph node with antibodies to (a) Leucocyte common antigen (LCA) (b) Cytokeratin (c) IgD heavy chain (d) T lymphocyte antigen (CD2). The anaplastic tumour cells (large cells, immunoblasts) clearly stain for LCA, Cytokeratin and IgD (staining for Lambda light chains was identical to IgD). The pan T cell antibody CD2 (Dako T11) stains only the scattered residual small lymphocytes, which also stain strongly for LCA in (a). The large tumour cells are unstained by the CD2 antibody. (Mag. (a) and (d) ×450, (b) and (c) ×720).

this is not unprecedented. Epithelial membrane antigen has been shown to be expressed by a small percentage of lymphomas/myeloma (Delsol *et al.*, 1984); lymphoid malignancies have been documented which express myeloid markers (Smith *et al.*, 1983). Even at the genotypic level using cDNA probes to Ig and T cell receptor genes, a small percentage of lymphoid tumours can be shown to have both T cell and Ig gene rearrangements in the same cells (Pelicci *et al.*, 1985).

Clearly by clinical and laboratory results our patient has myeloma. Whether the Cytok positivity of his tumour cells merely reflect an isolated malignant aberrant gene expression or may have biological/clinical significance and/or a particular association with IgD tumours must await further investigations and observations.

Finally, when investigating anaplastic tumours by immunophenotyping it is important that both antibodies (LCA and Cytok) be used. It should be stressed that this unusual case does not undermine the established usefulness of these antibody markers in the differential diagnosis of anaplastic tumours.

### References

GATTER, K.C., ALCOCK, C., HERYET, A. & MASON, D.Y. (1985). Clinical importance of analysing malignant tumours of uncertain origin with immuno-histological techniques. *Lancet*, **i**, 1302.

JEFFERIS, R. & MATHEWS, J.B. (1977). Studies of IgD myeloma proteins. Proteolytic digestion patterns. *Immunochem.*, **14**, 171.

DELSOL, G., GATTER, K.C., STEIN, H. & 4 others (1984). Human lymphoid cells may express epithelial membrane antigens. Implications for the diagnosis of human neoplasms. *Lancet*, **ii**, 1124.

SMITH, L.J., CURTIS, J.E., MESSMER, H.A., SENN, J.S., FURTHMAYR, H. & McCULLOCH, G.A. (1983). Lineage infidelity in acute leukemia. *Blood*, **61**, 1138.

PELICCI, P.G., KNOWLES, D.M. & FAVERA, R.D. (1985). Lymphoid tumours displaying rearrangements of both immunoglobulin and T cell receptor genes. *J. Exp. Med.*, **162**, 1010.